# β2 Adrenergic Regulation of the Phagocytic and Microbicide Capacity of Circulating Monocytes: Influence of Obesity and Exercise

**DOI:** 10.3390/nu12051438

**Published:** 2020-05-16

**Authors:** Isabel Gálvez, Leticia Martín-Cordero, María Dolores Hinchado, Eduardo Ortega

**Affiliations:** 1 Grupo de Investigación en Inmunofisiología, Departamento de Enfermería, Facultad de Medicina, Universidad de Extremadura, 06071 Badajoz, Spain; igalvez@unex.es; 2 Instituto Universitario de Investigación Biosanitaria de Extremadura (INUBE), 06071 Badajoz, Spain; leticiamartin@unex.es (L.M.-C.); mhinsan@unex.es (M.D.H.); 3 Grupo de Investigación en Inmunofisiología, Departamento de Enfermería, Centro Universitario de Plasencia, Universidad de Extremadura, 10600 Plasencia, Spain; 4 Grupo de Investigación en Inmunofisiología, Departamento de Fisiología, Facultad de Ciencias, Universidad de Extremadura, 06071 Badajoz, Spain

**Keywords:** β2 adrenergic receptors, terbutaline, obesity, exercise, monocytes, phagocytosis, microbicide activity, toll-like receptors, TLR4, TLR2

## Abstract

Obese individuals present anomalous immune/inflammatory responses with dysregulations in neuroendocrine responses and immune/stress feedback mechanisms. In this context, exercise and β2 adrenergic activation present monocyte-mediated anti-inflammatory effects that are modulated by obesity. However, these anti-inflammatory effects could immunocompromise the monocyte-mediated innate response against a pathogen challenge. Thus, the objective of this work was to evaluate the effect of obesity, and exercise in this condition, on the β2 adrenergic regulation of the phagocytic and microbicide capacity of circulating monocytes. C57BL/6J mice were allocated to different sedentary or exercised, lean or obese groups. Obese mice showed a lower monocyte-mediated innate response than that of lean mice. Globally, selective β2 adrenergic receptor agonist terbutaline decreased the innate response of monocytes from lean and obese sedentary animals, whereas exercise stimulated it. Exercise modulates β2 adrenergic regulation of the innate response in lean and obese animals, with a global stimulatory or neutral effect, thus abolishing the inhibitory effect of terbutaline occurring in sedentary animals. These effects cannot be explained only by changes in the surface expression of toll-like receptors. Therefore, in general, terbutaline does not hinder the effects of regular exercise, but regular exercise does abolish the effects of terbutaline in sedentary individuals.

## 1. Introduction

Obesity is a complex and multifactorial disease that results from a combination of genetic, epigenetic, and environmental factors, with diet and physical exercise generally being the main factors involved in its aetiology [1,2]. This condition is linked to the development of chronic comorbid conditions, especially the cluster of metabolic disorders (a combination of obesity, hypertension, hyperglycaemia, and dyslipidaemia, with increased risk of cardiovascular events) known as metabolic syndrome [2,3].

Obesity promotes chronic low-grade inflammation [4,5,6,7,8], a systemic condition characterised by increased systemic levels of some inflammatory cytokines (TNF-α, IL-1β, IL-6) with other inflammatory mediators [9]. It was recently reported that circulating monocytes from obese individuals are in a proinflammatory state, presenting a proinflammatory phenotype and activity profile [10]. The trigger for this inflammation is uncertain, and the causal relationship between obesity-induced low-grade inflammation and metabolic syndrome is not fully known [9,11,12,13,14].

Furthermore, the function of the hypothalamic–pituitary–adrenal (HPA) axis and the sympathetic nervous system (SNS) is also altered in obesity, leading to dysregulations in neuroendocrine responses and immune/stress feedback mechanisms [15,16,17,18,19]. Noradrenergic dysfunction, including SNS overactivity, is another characteristic of obesity and metabolic syndrome, contributing to the pathophysiology and development of the condition [20,21]. Adrenergic agonists, such as catecholamines secreted by the SNS and the adrenal glands, are implicated in metabolism regulation and most of the mechanisms of the immune response, including the innate response, and systemic and local release of inflammatory cytokines [22,23,24,25,26,27]. It is well known that both obesity and exercise affect the innate/inflammatory immune response [18,19,28,29], and that neuroimmunomodulation mainly participates in these responses through catecholamines [18,30].

Monocytes and other immune cells present β2 adrenergic receptors (AR) for adrenergic agonists [31,32,33]. The effect of β2 AR activation on monocytes/macrophages is usually anti-inflammatory and immunosuppressive, although under certain conditions, it can stimulate the innate response and result in proinflammatory effects [10,27,34,35,36]. In this context, obesity seems to be a factor that modulates the response of immune cells to β2 AR activation [35,36].

The beneficial effects of exercise are considered to generally be exerted through its anti-inflammatory effects by increasing catecholamine levels (due to activation of the HPA axis and the SNS) and potentially decreasing the percentage of cells with an inflammatory profile [35,37]. Physical exercise seems to be another factor that influences the β2 adrenergic regulation of inflammatory processes in immune cells [28,29], including monocytes [35,38]. Indeed, recent results from our group showed that β2 adrenergic stimulation in monocytes and macrophages leads to different inflammatory/innate responses in exercised animals compared to sedentary ones [35,36].

However, the β2 adrenergic regulation of the innate immune response (phagocytosis and microbicide capacity) of monocytes has not yet been completely elucidated; the influence of obesity in this regulation is also still not well-understood. Moreover, the effects of physical exercise (as a nonpharmacological strategy for obesity [27]) on the mechanisms underlying this regulation in obesity are still completely unknown.

Considering that the effect of obesity on this β2 adrenergic regulation could also be modulated by exercise, and that β2 adrenergic inhibition of the inflammatory response could be accompanied by immunosuppression of the phagocytic/microbicide response of monocytes, the objective of the present study was to determine the effect of obesity and exercise in this pathophysiological condition on the β2 adrenergic regulation of the phagocytic and microbicide capacity of circulating monocytes from C57BL/6J mice.

## 2. Materials and Methods

### 2.1. Animals and Experiment Design

**Figure 1:**
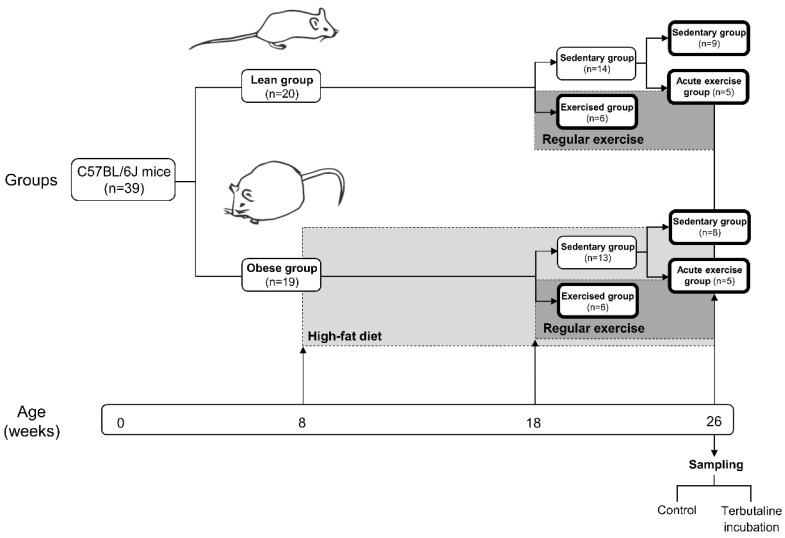
Schematic diagram of experiment study design showing mice groups, dietary and exercise interventions, chronogram, and sample treatment. Figure from Gálvez et al., *Nutrients* **2019**, *11*(11), 2630 [35].

Following the same protocol as in previous studies [35] (Figure 1), C57BL/6J mice were housed and bred in the animal facilities of the University of Extremadura from stock originally obtained from Envigo (Huntingdon, UK). At eight weeks of age, 39 mice were randomly allocated to different diets; one of the groups (*n* = 19, obese group) was placed on a high-fat diet (HFD) with 36% fat (58.8% of energy from fat) (260HF diet; SAFE, Augy, France), and the other group (*n* = 20, lean group) was placed on standard laboratory rodent chow (SD) (A04 diet; SAFE, Augy, France) containing 3.1% fat (8.4% of energy from fat), constituting the healthy control group.

After 10 weeks of the diet protocol, mice from both the lean and obese groups were randomly allocated to either a sedentary or a trained (performing regular exercise) group. The sedentary groups (lean sedentary group *n* = 14, obese sedentary group *n* = 13) did not perform regular physical exercise, whereas the exercised groups (lean regular exercise group *n* = 6, obese regular exercise group *n* = 6) underwent an 8-week program of regular exercise. Mice were 26 weeks old when the protocol finished. Just before sacrifice, mice from both the lean and obese sedentary groups were randomly allocated to either continuing in the corresponding sedentary group (lean sedentary group *n* = 9, obese sedentary group *n* = 8) or an acute exercise group (acute exercise lean group *n* = 5, acute exercise obese group *n* = 5). Acute exercise groups performed an acute bout of exercise immediately before sacrifice. Blood collection took place in anaesthetised animals following a 12 h fast. All evaluated parameters were determined in each animal.

During all experiment protocols, mice were housed individually, with free access to food and water throughout the study in order to quantify individual food consumption. Cages were kept in a temperature- and humidity-controlled room (temperature, 22 ± 1 °C; humidity, 60% ± 5%) and exposed to a 12/12 h light/dark cycle. Weekly measurements of body weight and food consumption were made. Food consumption was determined by weighing the total amount of food given at the start of each week and then subtracting by the amount of food remaining at the end of the week. The study was approved by the Bioethics Committee for Animal Experimentation of the University of Extremadura (registry number 115/2015), in accordance with the ARRIVE guidelines, and the national and European legislation for the protection of animals used for research.

### 2.2. Exercise Protocol

The regular and acute regular exercise protocols were described in previous works from our group [35,36]. The regular exercise program began when mice were 18 weeks old. It consisted of running on a treadmill (model 800, IITC Life Science Inc., Los Angeles, CA, USA), with no slope, and with duration and intensity adaptation, progression, and maintenance phases. The protocol was performed for 8 weeks, 3 days per week, during the mice’s active period (dark, 11:00–23:00 h). Regular exercise sessions progressed from 10 m/min for 10 min in the first week to 18 m/min for 45 min in the last week. This protocol was accepted to be able to induce physiological adaptations in mice [39,40]. Mice were sacrificed 72 h after the last training in order to avoid the evaluation of the acute effects of exercise.

The bout of acute exercise consisted of running on the treadmill for 5 min at 10 m/min followed by a progressive increase to 16 m/min for 35 min, with no slope, in the mice’s active period. Animals were sacrificed and samples collected immediately after the session.

### 2.3. Anaesthesia, Whole Blood Collection, Fasting Glucose, and Lipid-Profile Determination

Mice were anaesthetised with isoflurane (induction dose 3%–5%, maintenance dose 1.5%–3%). Whole blood was drawn by cardiac puncture, and 50 µL was used for the determination of the lipid profile (total cholesterol (TC), high-density lipoprotein cholesterol (HDL-C), calculated low-density lipoprotein cholesterol (cLDL-C), and triglycerides (TG)) and fasting glucose levels via portable analytical device Lux (Biochemical Systems International Srl, Arezzo, Italy).

### 2.4. Effect of Terbutaline on Monocyte Innate Function

As previously described in recent works from our group [35], whole blood was diluted in RPMI 1640 complete medium (Thermo Fisher Scientific, Waltham, MA, USA) except FBS. Samples of whole diluted blood were incubated in the presence or absence of 1 μM terbutaline (selective β2 AR agonist) (Sigma-Aldrich, St. Louis, MO, USA), with or without 5 μM butaxamine (β2-selective blocker) (Sigma-Aldrich MerkMillipore, Germany) in order to check the effect of terbutaline. Following 5 h incubation at 37 °C, 5% CO_2_, samples were centrifuged, and pellets were either incubated in staining buffer (600 µL of cold PBS solution + 0.5% bovine serum albumin (BSA) + 2 mM ethylenediaminetetraacetic acid (EDTA) (Thermo Fisher Scientific, Waltham, MA, USA)) to obtain cells for antibody staining, or in PBS solution + 2% FBS (100 µL) for phagocytosis and oxidative-burst assays.

#### 2.4.1. Phagocytic and Oxidative-Burst Assays via Flow Cytometry

Phagocytosis and microbicide capacity of opsonised bacteria were evaluated in circulating monocytes by flow cytometry, obtaining very accurate determinations of the ability of monocytes to ingest bacteria and produce superoxide anion (O2–, reflecting oxygen-dependent microbicide activity). Briefly, opsonised bacteria (*Escherichia coli*) were fixed and stained with fluorescein isothiocyanate (FITC) (final concentration 30 μg/mL). Cells were incubated with *E. coli* FITC, Hoechst 33342 (10 μg/mL), hydroethidine (HE; 10 μM) to detect the intracellular production of superoxide anions by NADPH oxidase, and To-Pro-3 (0.1 μM) diluted in PBS + 2% FBS. Sample analysis was performed via flow cytometer (MACSQuant 10, Miltenyi Biotech, Germany), and data were analysed with MACSQuantify (Miltenyi Biotech) and Flowlogic (Inivai, Australia) software. Monocyte population was gated by FSC/SSC parameters. Results are expressed as the percentage of “phagocytic monocytes” (monocytes CFSE+) or “microbicidal monocytes” (monocytes HE+), and as measurements of phagocytic or oxidative activity (mean fluorescence intensity, MFI) [36].

#### 2.4.2. Toll-Like Receptor 2 (TLR2) and 4 (TLR4) Surface Expression via Flow Cytometry

Cells were fixed by diluting cells in a staining buffer (600 μL) and Inside Fix reagent (750 μL) from the Inside Stain Kit (Miltenyi Biotec, Bergisch Gladbach, Germany). Cells were then centrifuged and washed in staining buffer (300 μL), and kept overnight at 4 °C. After another centrifugation, Inside Perm reagent (50 µL) from the Inside Stain Kit was added to pellets. Next, cells were incubated with conjugated antibody CD282 (TLR2)-APC and CD284-PE (3 µg per 10^6^ cells) (Miltenyi Biotec). Samples were finally washed and fixed in order to perform analysis using flow cytometer CytoFLEX S (Beckman Coulter Life Sciences, Indianapolis, IN, USA). CytExpert software (Beckman Coulter Life Sciences, Indianapolis, IN, USA) was used to process data on the basis of the monocyte population gated by FSC/SSC parameters [35].

### 2.5. Statistical Analysis

Values are expressed as the mean ± standard error of the mean (SEM). The normal distribution of the variables was checked using the Kolmogorov–Smirnov normality test, followed by Student’s *t* test for comparisons between two groups. The minimal significance level was set at *p* < 0.05. Statistical analyses were performed with GraphPad Prism 7.0 (GraphPad Software Inc., San Diego, CA, USA).

## 3. Results

### 3.1. Body Weight, Dietary Intake, Fasting Blood Glucose, and Lipid Profile—Effect of Exercise

As expected, at the end of the high-fat-diet protocol, obese groups presented significantly higher body weight than that of animals fed standard chow, even with HFD animals ingesting less food than the control group. The obese sedentary group showed significantly higher levels of glucose, TG, TC, HDL-C, and cLDL-C than those of the lean sedentary group (Table 1). These results corroborate that this a good HFD protocol for inducing an experimental model of obesity and obesity-associated metabolic dysregulation in C57BL/6J mice, as previously published in a recent article from our group [35].

After the exercise protocol, obese mice performing regular exercise presented a lower body weight than that of their corresponding sedentary group. Even though a trend towards lower fasting glucose levels was observed, neither lean nor obese animals that underwent the program of habitual exercise showed significant differences in glucose levels with respect to their corresponding sedentary group. Both lean and obese mice performing regular exercise presented higher levels of HDL-C, and lower cLDL-C and TG levels than those of their corresponding sedentary group. Thus, lipid profile improved in both lean and obese animals after habitual exercise, but not after acute exercise (Table 1). These data confirmed that the protocol of regular exercise is optimal for evaluating exercise-induced physiological and metabolic adaptations in HFD-induced obese mice.

### 3.2. Effect of β2 Adrenergic Activation by Terbutaline on Phagocytic and Microbicide Capacities of Circulating Monocytes from Lean and Obese Mice

**Figure 2:**
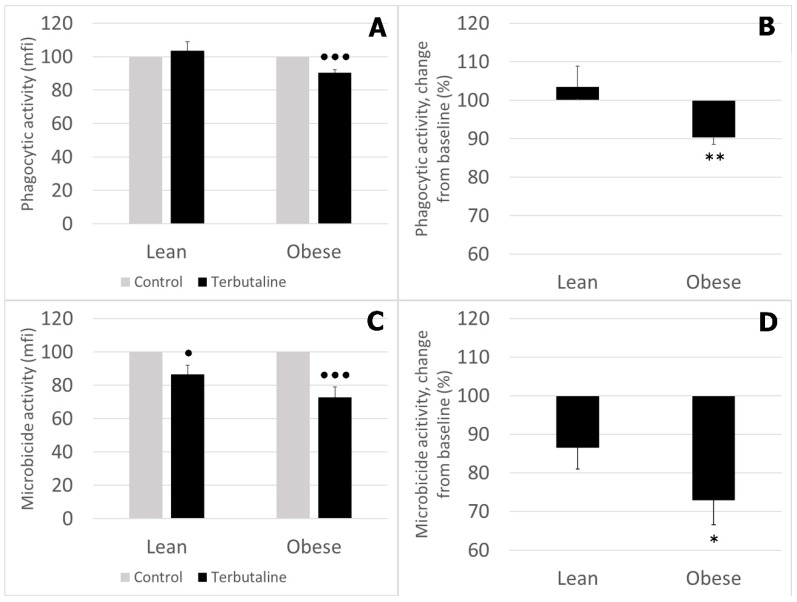
Effect of β2 adrenergic agonist terbutaline on (**A,B**) phagocytic and (**C,D**) microbicide activity of circulating monocytes from lean and obese mice, expressed as the percentage change from baseline (giving “100” to control values in the absence of terbutaline). Columns represent the mean ± SEM of independent assays performed in duplicate. * *p* < 0.05 and ** *p* < 0.01 vs. corresponding lean value; • *p* < 0.05 and ●●● *p* < 0.001 vs. corresponding control value.

Figure 2 shows results corresponding to the effect of terbutaline on the phagocytic and microbicide activities of monocytes in lean and obese animals. These results are expressed and statistically analysed in percentage change from baseline, giving “100” to the basal values (in the absence of β2 adrenergic stimulation).

β2 adrenergic stimulation by terbutaline significantly decreased the phagocytic activity (Figure 2A) of monocytes from obese mice, and decreased the microbicide activity (Figure 2C) of monocytes from lean and obese mice. This effect was significantly different in obese vs. lean animals (Figure 2B,D).

The results and statistical study of the effect of terbutaline and butaxamine on the percentage of monocytes presenting phagocytic and microbicide activities are shown in Table 2. Percentage of phagocytic monocytes was significantly lower in obese mice. With β2 AR stimulation, phagocytic percentage decreased in lean and obese mice. The percentage of microbicidal monocytes was also lower in obese than that in lean mine. Terbutaline only caused a decrease in this microbicide percentage in obese animals. The presence of butaxamine (β2 AR blocker) abolished these effects, confirming the β2 selective effect of terbutaline in this immunological context as well.

### 3.3. Effect of Exercise in β2 Adrenergic Regulation of Phagocytic and Microbicide Capacity of Circulating Monocytes from Obese and Lean Mice

**Figure 3:**
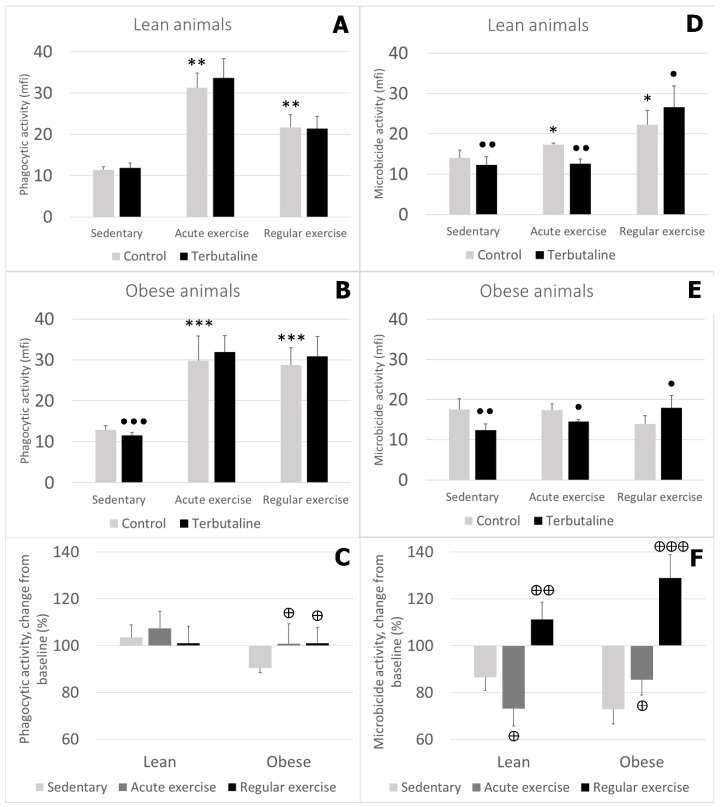
Effect of β2 adrenergic agonist terbutaline on (**A–C**) phagocytic and (**D–F**) microbicide activity of circulating monocytes from (**A,D**) lean and (**B,E**) obese mice (sedentary, and acute and regular exercise); effect of terbutaline expressed as percentage change from (**C,F**) baseline, giving “100” to control values in terbutaline absence. Columns represent mean ± SEM of independent assays performed in duplicate. * *p* < 0.05, ** *p* < 0.01, and *** *p* < 0.001 vs. corresponding control sedentary value; • *p* < 0.05, ●● *p* < 0.01, and ●●● *p* < 0.001 vs. corresponding control value; ⊕ *p* < 0.05, ⊕⊕ *p* < 0.01, ⊕⊕⊕ *p* < 0.001 vs. corresponding sedentary value.

Monocytes from animals performing acute or regular exercise showed higher phagocytic activity than those from sedentary control groups, in both healthy lean (Figure 3A) and obese (Figure 3B) animals. Monocytes’ microbicide activity was also higher after acute or regular exercise, but only in lean animals (Figure 3D). Obese animals did not show any significant change in their monocytes’ microbicide activity with exercise (Figure 3E).

β2 adrenergic stimulation by terbutaline only caused a decrease in the phagocytic activity of monocytes from sedentary obese animals, and there were no changes in the exercised groups (Figure 3B). Terbutaline did not cause any significant effect in the phagocytic activity of monocytes from lean animals, either sedentary or exercised (Figure 3A). Additionally, when expressing these results in percentage change from the baseline, we observed that monocytes’ response to terbutaline in lean sedentary, acute-, and regular-exercise groups was similar, whereas this response was significantly different in the obese groups: decreased activity in obese sedentary mice vs. no change in exercised groups (Figure 3C).

β2 adrenergic stimulation reduced monocytes’ microbicide activity in the sedentary and acute-exercise groups in both lean and obese mice. However, terbutaline increased the microbicide activity of monocytes from lean and obese animals performing regular exercise (Figure 3D,E). Expressed in percentage change from the baseline, data showed that there was a similar behaviour in lean and obese mice: changes in monocytes’ microbicide activity in response to β2 adrenergic stimulation were significantly different in the exercised groups compared to the sedentary groups, both in lean and obese animals (Figure 3F).

**Figure 4:**
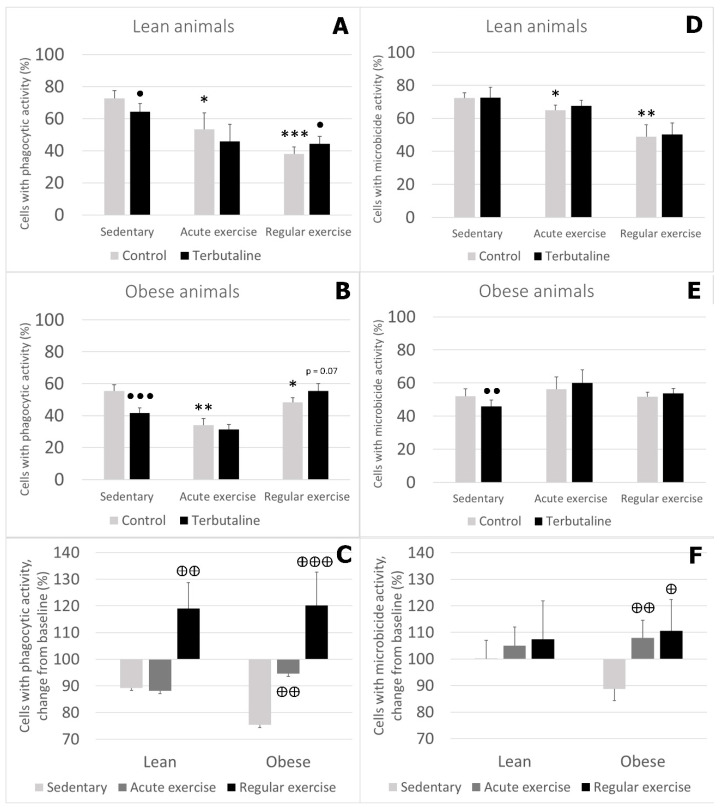
Effect of the β2 adrenergic agonist terbutaline on percentage of monocytes with (**A–C**) phagocytic and (**D–F**) microbicide activity from lean (**A,D**) and obese (**B,E**) mice (sedentary, an acute and regular exercise); effect of terbutaline expressed as percentage change from baseline (**C,F**), giving “100” to control values in terbutaline absence. Columns represent mean ± SEM of independent assays performed in duplicate. * *p* < 0.05, ** *p* < 0.01, and *** *p* < 0.001 vs. corresponding control sedentary value; • *p* < 0.05, ●● *p* < 0.01, and ●●● *p* < 0.001 vs. corresponding control value; ⊕ *p* < 0.05, ⊕⊕ *p* < 0.01, and ⊕⊕⊕ *p* < 0.001 vs. corresponding sedentary value.

Regarding the percentage of cells showing these phagocytic/microbicide activities, the percentage of monocytes with phagocytic activity was lower in lean and obese animals performing acute or regular exercise (Figure 4A,B), and the percentage of cells with microbicide activity was only lower in lean mice performing acute or regular exercise, but not in obese mice (Figure 4D,E).

Terbutaline decreased the percentage of monocytes with phagocytic activity in both lean and obese sedentary mice, and increased it in lean and obese animals performing regular exercise (with statistical significance only in lean mice). There were no significant changes in animals performing acute exercise (Figure 4A,B). When we represented these results as percentage change from the baseline, there was a significantly different response to terbutaline in the regular-exercise groups compared to the sedentary ones, and this behaviour was observed in both lean and obese mice (Figure 4C).

β2 adrenergic stimulation by terbutaline only caused a decrease in the percentage of cells with microbicide activity in obese sedentary mice (Figure 4E). No statistically significant changes were observed in any of the exercised groups. Analysed as percentage change from the baseline, the response to terbutaline was significantly different in obese exercised groups compared to the obese sedentary group, but this behaviour did not occur in lean mice (with a similar response to terbutaline in all groups) (Figure 4F).

### 3.4. Effect of Exercise in β2 Adrenergic Regulation of TLR2 and TLR4 Expression in Circulating Monocytes from Obese and Lean Mice

Obese animals’ monocytes showed lower TLR2 and higher TLR4 expression compared to those of lean mice.

Lean animals performing acute or regular exercise presented lower TLR2 expression values, whereas TLR4 values had no significant changes. Obese animals performing acute but not regular exercise presented significantly higher expression of TLR2. Regarding TLR4, both acute and regular exercise caused a reduction in its expression in obese mice.

β2 adrenergic stimulation with terbutaline only caused a decrease in TLR2 expression in lean sedentary animals, but there were no significant changes in any obese or exercised group. However, TLR4 expression values decreased in both lean and obese sedentary mice with β2 adrenergic stimulation. Statistically significant changes in TLR4 expression values after β2 adrenergic stimulation in exercised animals were only observed in lean mice, with an increase in TLR4 with regular exercise, and a decrease with acute exercise (Table 3).

## 4. Discussion

The present investigation is the first to analyse the influence of obesity and exercise in individuals with this condition on β2 adrenergic regulation of the phagocytic and microbicide capacity of monocytes.

Obese sedentary animals presented a lower percentage of monocytes with phagocytic and microbicide activity than that of lean ones. This could be indicative of reduced innate immune response against pathogens in obese individuals that has even been linked to a higher risk of infection, as has been reported in previous studies in humans and animal models [17,41,42,43,44]. Interestingly, recent results from our laboratory regarding the innate function of macrophages did not reveal a lower phagocytic/microbicide capacity in the same model of HFD-induced obese mice [36], showing that obesity-induced changes in the innate response of phagocytes depend on the tissue location and differentiation status of cells.

The effect of β2 AR stimulation in monocytes from sedentary animals was globally inhibitory in lean and obese mice, a similar behaviour to that previously observed in leukocytes [45] and particularly in macrophages [36]. The percentage of phagocytic monocytes decreased in lean and obese animals after incubation with terbutaline, whereas the percentage of microbicidal monocytes only decreased in obese mice, in accordance with the behaviour observed in macrophages [36]. β2 AR stimulation also caused a decrease in monocytes’ phagocytic activity (only in obese mice) and microbicide activity (in both lean and obese animals). In previous studies in macrophages, terbutaline caused a decrease in phagocytic and microbicide activity in both lean and obese mice, so the inhibitory effect was not modified by obesity [36]. The present results could indicate that, while both the phagocytic/microbicide percentage and activity of monocytes decrease after β2 AR stimulation in obese animals, there seems to be an attempt of compensation for this decrease in lean animals: the percentage of phagocytic monocytes decreases, but activity does not, and the percentage of microbicidal monocytes remains unchanged, while their microbicide activity decreases. Furthermore, when analysing these reductions in innate capacity in percentage change from the baseline, they were significantly greater in obese animals than those in lean ones. This could be reflecting a higher impairment of innate function in obese than in lean animals in response to β2 AR stimulation by terbutaline despite the overall inhibitory effect in both groups. Moreover, these results further confirm that the inhibitory effect of adrenergic agonists on the innate function of phagocytes is, at least partly, selectively mediated by β AR [36], while stimulatory effects were shown to need the participation of α AR [24,46]. In this way, strategies aimed at regulating the inflammatory response of phagocytes in obesity, both nonpharmacological, such as exercise [37], and pharmacological, such as β2 AR, agonists [47,48] (alone or combined) should be studied in order to determine optimal conditions, limitations, and avoid potential immunosuppression in obese individuals.

Some studies reported the stimulatory effect of exercise on phagocyte-mediated (including monocytes) innate immune function in healthy lean individuals [28,49,50]. Regarding the influence of obesity in the effect of exercise on the monocyte-mediated innate response, there was a general stimulation of innate activity with physiological compensation between innate activity and the percentage of cells presenting this activity. For example, after performing acute or regular exercise, the percentage of monocytes with phagocytic activity was lower, but mean phagocytic activity in the cell was greater both in lean and obese mice, thus compensating for the reduced number of cells with this capacity. In accordance with this observed exercise-induced stimulation of the phagocytic function of monocytes, previous results from our group also showed that, in different animal models including models of obesity, exercise stimulates the phagocytic capacity of macrophages [17,41,50,51,52]. However, the percentage of microbicidal monocytes was lower, and microbicide activity greater only in lean animals performing exercise compared to sedentary ones; neither of these parameters changed in obese animals, thus showing that obesity influences the effect of exercise in the context of microbicide function (resulting in an absence of stimulation). These results are, in general terms, in agreement with those observed in macrophages from the same animal model [36].

The key question is if exercise modifies the β2 adrenergic regulation of the innate immune response of monocytes, and if this potential influence on the β2 adrenergic regulation is different when exercise is performed by obese individuals.

With respect to phagocytosis, in general, β2 adrenergic stimulation caused a global increase of phagocytosis in monocytes from lean and obese animals after regular exercise. Our results showed that β2 adrenergic regulation of the percentage of monocytes with phagocytic activity was significantly different in animals performing regular exercise compared to sedentary animals. While this parameter was inhibited both in lean and obese sedentary animals in response to β2 adrenergic activation, it increased when these animals had been subjected to regular exercise, thus abolishing the inhibitory effect of terbutaline in sedentary animals. This response pattern was similar in obese and lean animals. Regarding phagocytic activity, monocytes from none of the groups of lean mice showed changes in response to terbutaline, so β2 adrenergic regulation was the same regardless of the performance of exercise. On the other hand, the β2 adrenergic regulation of phagocytic activity of monocytes from obese mice was different in sedentary (inhibition) and exercised animals (no significant changes and tendency towards stimulation, thus abolishing inhibition occurring in sedentary mice in response to terbutaline). In this way, the stimulation of phagocytic activity caused by exercise was not impaired by potential β2 adrenergic-induced inhibition, neither in lean nor obese animals.

Regarding microbicide capacity, β2 adrenergic regulation globally increased monocytes’ microbicide capacity in lean and obese mice after regular exercise. The percentage of monocytes with microbicide activity remained unchanged in lean exercised animals in the same way as in sedentary animals in response to terbutaline. In obese mice the, response to terbutaline in exercised animals was similar to that in lean exercised mice, since the percentage of monocytes with microbicide activity remained unchanged as well. However, in this case, this β2 adrenergic regulation was different to that in obese sedentary mice (inhibition), so exercise abolished inhibition caused by terbutaline in sedentary mice. On the other hand, β2 adrenergic activation increased microbicide activity in monocytes from lean and obese animals that underwent the regular-exercise protocol, thus abolishing inhibition caused in sedentary and acute-exercise animals. Consequently, regular exercise modified the β2 adrenergic regulation of microbicide activity in lean and obese individuals.

Evaluating all these results regarding β2 adrenergic regulation of phagocytosis and microbicide capacity in exercised animals as a global response, there was general stimulation (in the percentage of phagocytic monocytes and their microbicide activity) or no changes, particularly with regular exercise. This regulation was different to that in sedentary animals, especially (and always) in obese mice, mostly abolishing or reverting the inhibitory effect of β2 adrenergic activation occurring in sedentary animals. This behaviour was similar in lean and obese mice. Therefore, exercise caused adaptation in the response of monocytes to terbutaline by inducing a stimulatory effect in their innate response, as has been reported in peritoneal macrophages from the same animal model of obesity [36]. In macrophages, obesity hindered this immunophysiological adaptation to regular exercise [36]; in monocytes, not only did this not occur, but the response was also even stronger in obesity. Moreover, while acute exercise did not modify the inhibitory capacity of terbutaline in macrophages [36], acute exercise did abolish this effect in some of the analysed parameters of the innate function of monocytes, but not all of them, with this behaviour being clearer and stronger with regular exercise. To the best of our knowledge, this work is the first to determine the effect of exercise in lean and obese individuals on the β2 adrenergic regulation of the innate function of monocytes, so we cannot discuss these results in relation to similar investigations focusing on this cell type or other phagocytes. In fact, a recent review of the literature revealed the lack of investigations in this field [27]. Although it could be plausible speculation, we cannot conclude that differential responses to terbutaline and exercise depend, at least solely, on changes in the expression and internalisation of β2 AR in monocytes, since in our previous studies, we determined no significant differences in the same animals in the monocytes’ membrane surface expression of β2 AR between lean and obese animals (including in their response to physical exercise) [35]. Additionally, catecholamines and other β2 AR agonists can inhibit interferon gamma (IFN-γ) production by Th1 cells [23]. Since whole blood samples incubated with terbutaline contained monocytes and also lymphocytes, it could be speculated that the inhibitory effects of phagocytic and microbicide capacity could also be indirectly mediated by the previous inhibition of IFN-γ release by Th1 cells, since it is well known that this cytokine can activate Fc receptor-mediated innate response in monocytes/macrophages [53,54]. Nevertheless, under certain pathological conditions, IFN-γ can inhibit Fc receptor-mediated macrophage function, for example, the phagocytosis of tumor cells [55].

Examining the findings of the present work in the light of previous results regarding the β2 adrenergic regulation of inflammation in monocytes from the same animal model of obesity and exercise, we come to the conclusion that anti-inflammatory effects could be accompanied by a decrease in innate function, so it is imperative to be careful with respect to the potential use of β2 adrenergic agonists as a strategy against inflammation in obesity in order to avoid an increased susceptibility to infection, especially when they are combined with other strategies such as exercise [27,47,48].

Toll-like receptors (TLRs) participate in the modulation of inflammation and innate response. Moreover, TLRs, especially TLR2 and TLR4, induce insulin resistance, which is crucial in the pathogenesis of obesity [56]. Although the TLR4 signalling pathway is also involved in the phagocytic response, it is acknowledged as one of the main triggers of the obesity-induced inflammatory response [56,57], in accordance with the elevated percentage of monocytes from obese animals expressing TLR4 observed in the present study. Regarding TLR expression in our animal model, in general, the lower expression of TLR2 and higher of TLR4 in obese animals could explain the observed lower phagocytic capacity of monocytes from obese mice, as well as their higher inflammatory activity [10], since TLR2 has been mostly associated with the phagocytic response (as one of the strongest inducers of this process) [58,59,60] while TLR4 is linked to the release of inflammatory mediators [57,61], being one of the weakest inducers of phagocytosis compared to other TLRs [60]. In this context, the decrease in the percentage of monocytes presenting terbutaline-induced phagocytic activity (in sedentary subjects) seems to be related to the decrease in the percentage of monocytes expressing TLR2 and TLR4, which could also explain the anti-inflammatory effects of terbutaline in these cells, particularly in obese individuals [10].

Some of the variations in phagocytosis in response to exercise could also be clarified by these changes in TLR expression. Moreover, the decrease in TLR4 expression in exercised obese animals could be related to the anti-inflammatory effects of exercise previously observed by our group in the same animal model of obesity [35]. This confirms previous investigations that proposed that the anti-inflammatory effects of exercise could be mediated by a reduction in the expression and/or activation of TLR4 in innate immune cells, with the subsequent inhibition of downstream inflammatory responses [37,62,63]. This mechanism is particularly important in obese subjects since it hinders one of the main inflammatory responses triggered by obesity, thus potentially contributing to the protective effect of exercise against insulin resistance and the prevention of the development of metabolic diseases [62]. However, there seems to be no consensus about the exact role of TLRs and the underlying mechanisms in this modulation.

β2 AR stimulation in sedentary mice caused similar effects to those of exercise. Furthermore, exercise abolished the inhibitory effects of β2 adrenergic stimulation: β2 AR stimulation did not cause a decrease in TLR2 or TLR4 expression in lean or obese exercised animals. Accordingly, terbutaline-mediated changes of the phagocytic capacity of monocytes from exercised animals were stimulatory. This is the first time that results regarding β2 adrenergic activation and TLRs are reported, particularly in the context of obesity and exercise.

Even though variations in TLR2 and TLR4 expression can explain some of the changes in the innate/inflammatory response mediated by terbutaline and exercise, not all of them seem to be completely related or dependent on this mechanism, in the same way that these changes can neither be explained by variations in the expression of β2 adrenoreceptors. Therefore, further studies focusing on molecular signalling mechanisms underlying or contributing to these responses in obesity and exercise in this condition are needed, including the evaluation of other adrenoreceptors (particularly β1 AR-mediated proinflammatory intracellular signalling [64]). Moreover, it is also interesting to elucidate TLR function in this context by evaluating the effects (such as cytokine production) of monocyte stimulation with TLR ligands.

## 5. Conclusions

The main and physiologically relevant conclusions of the present study are:

- Obese sedentary animals present a reduced monocyte-mediated innate immune response compared to that of lean sedentary animals.
- The effect of β2 adrenergic activation on the innate function of monocytes from sedentary animals was globally inhibitory in lean and obese mice, although this inhibition was significantly greater in obese animals, probably reflecting the higher impairment of innate function in obese animals in response to β2 AR stimulation by terbutaline.
- Exercise stimulates phagocytic function in lean and obese mice, and microbicide function only in lean mice. Therefore, the beneficial effects of exercise are stronger in lean animals.
- Exercise, especially regular exercise, modifies β2 adrenergic regulation of the innate response of monocytes from lean and obese mice, with a global stimulatory or neutral effect, thus abolishing the inhibitory effect occurring in sedentary animals. This behaviour was similar in lean and obese animals but was stronger in obese ones.
- Changes in TLR expression seem to partially explain some innate-/inflammatory-response variations related to obesity, exercise, and β2 AR stimulation.
- In general, terbutaline seems to not hinder the effects of regular exercise, but regular exercise does abolish the effects of terbutaline (different effects in sedentary vs. exercised).

Although further studies are clearly needed, these results can contribute to the better understanding of the adrenergic regulation of the innate immune function in monocytes, so that, ultimately, anti-inflammatory strategies such as physical exercise and β2 adrenergic agonists, both alone and combined, can be optimised for the treatment of systemic inflammation in obesity.

## Figures and Tables

**Table 1:** Body weight, dietary and energy intake, and metabolic parameters in lean and obese mice, without (sedentary) and with acute or regular exercise.

	Lean			Obese		
	Sedentary	Acute Exercise	Regular Exercise	Sedentary	Acute Exercise	Regular Exercise
Body weight (g)	29.28 ± 1.17	30.15 ± 2.49	25.7 ± 1.27	42.28 ± 1.15***	40.37 ± 2.93	36.14 ± 2.98●
Dietary intake (g/day)	3.96 ± 0.22	4.16 ± 0.09	4.05 ± 0.06	2.68 ± 0.11***	2.62 ± 0.08	2.49 ± 0.03●●
Energy intake (kJ/day)	55.32 ± 3.15	58.16 ± 1.38	56.63 ± 0.86	62.01 ± 2.67***	60.50 ± 1.90	57.63 ± 0.71●●
Glucose (mg/dL)	218.90 ± 13.26	174.45 ± 32.06●	196.37 ± 25.53	311.50 ± 30.93**	222.75 ± 23.12●	282.5 ± 27.85
Cholesterol (mg/dL)						
TC	103.69 ± 2.22	<99†	106.75 ± 2.90	172.70 ± 19.28***	175 ± 41.82	178.12 ± 24.68
HDL-C	42.15 ± 2.93	-	51.75 ± 3.91●	59.70 ± 5.69**	55.5 ± 2.72	75.25 ± 4.39●
cLDL-C	50.75 ± 3.49	-	39.4 ± 1.74●	88.83 ± 16.05*	78 ± 12	38.5 ± 1.5●
TG (mg/dL)	86.80 ± 1.86	88 ± 0.01	76.62 ± 0.73●	91.55 ± 1.99*	98.75 ± 7	80 ± 1.43●●●

Each value represents the mean ± SEM of independent assays performed in duplicate. * *p* < 0.05, ** *p* < 0.01, *** *p* < 0.001 vs. lean-sedentary-mice-group values; ● *p* < 0.05, ●● *p* < 0.01, ●●● *p* < 0.001 vs. corresponding sedentary-mice-group values. TC, total cholesterol; HDL-C, high-density lipoprotein cholesterol; cLDL-C, calculated low-density lipoprotein cholesterol; TG, triglycerides; †, below limit of detection. Table from Gálvez et al., *Nutrients* 2019, *11*(11), 2630 [35].

**Table 2:** Effect of β2 adrenergic agonist terbutaline and β2 adrenergic antagonist butaxamine on percentage of phagocytic monocytes and percentage of microbicidal monocytes from lean and obese mice.

		Control	Terbutaline	Terbutaline + Butaxamine
Phagocytic percentage (%)	Lean	72.54 ± 4.98	64.42 ± 4.98•	70.02 ± 6.43◊◊
	Obese	55.41 ± 3.95**	41.63 ± 3.26•••	48.85 ± 3.15◊
Microbicide percentage (%)	Lean	72.32 ± 3.07	72.60 ± 6.35	70.61 ± 5.95
	Obese	52.07 ± 4.48***	45.92 ± 3.90••	59.59 ± 4.05◊◊

Each value represents the mean ± SEM of independent assays performed in duplicate. ** *p* < 0.01 and *** *p* < 0.001 vs. corresponding control lean value; • *p* < 0.05, •• *p* < 0.01, and ••• *p* < 0.001 vs. corresponding control value (absence of terbutaline and butaxamine); ◊ *p* < 0.05 and ◊◊ *p* < 0.01 vs. corresponding terbutaline value.

**Table 3:** Effect of β2 adrenergic agonist terbutaline on TLR2 and TLR4 expression in circulating monocytes from lean and obese mice (sedentary, an acute and regular exercise).

		Without Terbutaline (Control)			With Terbutaline		
		Sedentary	Acute Exercise	Regular Exercise	Sedentary	Acute Exercise	Regular Exercise
TLR2+ monocytes (%)	Lean	78.35 ± 3.62	67.77 ± 1.36••	63.59 ± 6.21•	64.64 ± 6.07◊	66.09 ± 1.40	61.59 ± 6.84
	Obese	64.18 ± 8.01*	77.86 ± 1.41•	62.35 ± 7.78	64.57 ± 11.30	69.28 ± 8.90	63.91 ± 6.71
TLR4+ monocytes (%)	Lean	79.50 ± 5.10	88.58 ± 3.28	71.89 ± 4.87	55.35 ± 10.77◊	74.01 ± 5.10◊◊◊	83.35 ± 3.06◊
	Obese	91.16 ± 1.76**	85.26 ± 2.74•	76.53 ± 5.35••	67.33 ± 10.81◊	82.11 ± 1.45	79.66 ± 5.98

Each value represents mean ± SEM of independent assays performed in duplicate. * *p* < 0.05 and ** *p* < 0.01 vs. corresponding lean sedentary value; • *p* < 0.05 and •• *p* < 0.01 vs. corresponding sedentary control value; ◊ *p* < 0.05 vs. corresponding control value (absence of terbutaline).
